# Supplementary figures and images for: Testosterone and Long-Pulse-Width Stimulation (TLPS) on Denervated Muscles and Cardio-Metabolic Risk Factors After Spinal Cord Injury: A Pilot Randomized Trial
Source: Cells. 2025 Dec 11;14(24):1974. doi: 10.3390/cells14241974 (PMC12731934; doi:10.3390/cells14241974)

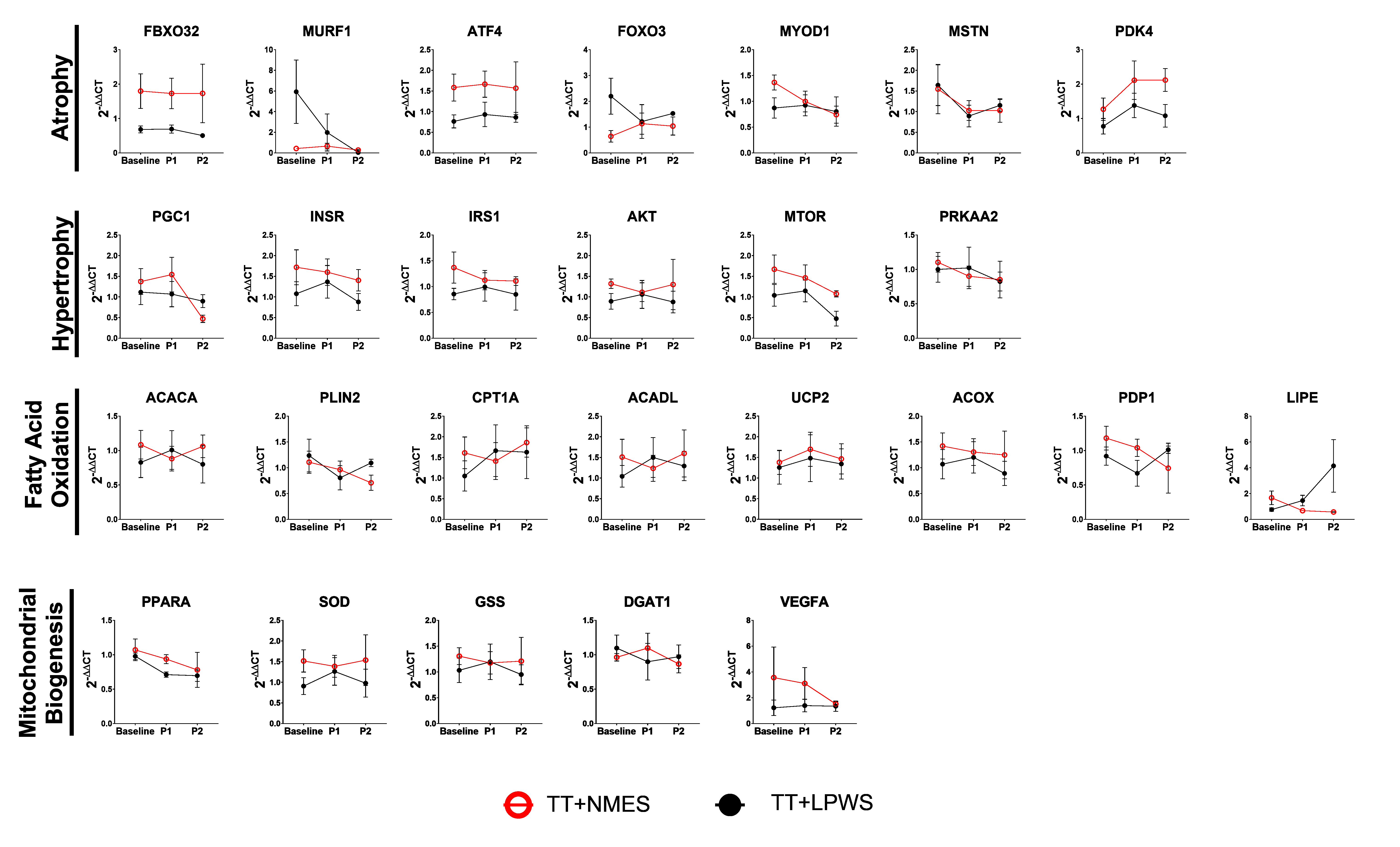

Supplement: Supplementary file 1 [file cells-14-01974-s001.zip › cells-3930921-supplementary.tif]
